# Effect of vitamin D3 on lipid droplet growth in adipocytes of mice with HFD‐induced obesity

**DOI:** 10.1002/fsn3.3618

**Published:** 2023-08-24

**Authors:** Jingjing Zhang, Yuanfan Zhang, Yong Zhou, Wenxin Zhao, Jialu Li, Dan Yang, Lian Xiang, Tingwan Du, Ling Ma

**Affiliations:** ^1^ Department of Clinical Nutrition Affiliated Hospital of Southwest Medical University Luzhou Sichuan China; ^2^ Department of Nutrition and Food Hygiene, School of Public Health Southwest Medical University Luzhou Sichuan China; ^3^ Department of Medical Cell Biology and Genetics, School of Basic Medical Science Southwest Medical University Luzhou Sichuan China

**Keywords:** high‐fat diet, lipid droplets, mice, obesity, VitD3

## Abstract

Vitamin D‐regulating action of PPARγ on obesity has been confirmed on adipocyte differentiation. However, it is not clear whether vitamin D affects the morphological size of mature adipocytes by influencing the expression of PPARγ in vivo. Our hypothesis was that Vitamin D3 (VitD3) inhibits the growth of adipocyte size by suppressing PPARγ expression in white adipocytes of obese mice. Five‐week‐old male C57BL/6J mice were randomly divided into normal diet and high‐fat diet groups. After 10 weeks, the body weight between the two groups differed by 26.91%. The obese mice were randomly divided into a high‐fat diet, solvent control, low‐dose VitD3 (5000 IU/kg·food), medium‐dose VitD3 (7500 IU/kg·food), high‐dose VitD3 (10,000 IU/kg·food), and PPAR γ antagonist group, and the intervention lasted for 8 weeks. Diet‐induced obesity (DIO) mice fed high‐dose VitD3 exacerbated markers of adiposity (body weight, fat mass, fat mass rate, size of white and brown adipocytes, mRNA, and protein levels of ATGL and Fsp27), and the protein level of ATGL and Fsp27 decreased in the low‐dose group. In conclusion, high‐dose VitD3 possibly via inhibiting the ATGL expression, thereby inhibiting lipolysis, increasing the volume of adipocytes, and decreasing their fat‐storing ability resulted in decreased Fsp27 expression. Therefore, long‐term high‐dose oral VitD3 may not necessarily improve obesity, and we need more clinical trials to explore the intervention dose and duration of VitD3 in the treatment of VitD3 deficiency in obese patients.

## Graphical abstract

High‐dose VitD3 possibly via inhibiting the ATGL expression, thereby inhibiting lipolysis, increasing the volume of adipocytes, and decreasing their fat‐storing ability resulted in decreased Fsp27 expression. Therefore, long‐term high‐dose oral VitD3 may not necessarily improve obesity, the intervention dose and duration of VitD3 in the treatment of VitD3 deficiency in obese patients are the key points.

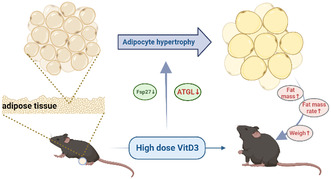

## 1 INTRODUCTION

According to the Global Burden of Disease 2015 Obesity Collaborators, the prevalence of obesity was 5.0% among children and 12.0% among adults worldwide. The prevalence of obesity increased between 1980 and 2015 (GBD 2015 Obesity Collaborators et al., 2017). Obesity, which is characterized by excessive fat accumulation, is a serious public health problem. White fat depot is the main type of adipose tissue in the body that stores energy and is a highly active endocrine organ involved in the development of various chronic diseases. Fat depots store energy in the form of triglycerides, and their development is chronic (Merkel et al., 2019). Adipose tissue accumulates through adipocyte hyperplasia, hypertrophy, or both. Weight gain in adults is mainly manifested as adipocyte hypertrophy. The size of adipocytes is determined by the dimensions of lipid droplets (LDs), whose growth is affected by factors such as lipogenesis, LD fusion, and lipolysis (Chapman et al., 2019).

LD consists of a core of neutral lipids encircled by a phospholipid monolayer that is embedded with integral and peripheral proteins, alternating between periods of growth and consumption through enzymatic hydrolysis mediated by lipases (lipolysis) or through a selective form of autophagy (lipophagy) (Olzmann & Carvalho, 2019). LDs serve diverse cellular functions, including isolating toxic lipids and acting as dynamic lipid storage sites that enable rapid mobilization of fatty acids for energy, membrane biosynthesis, and lipid signaling pathways (Bersuker & Olzmann, 2017; Jackson, 2019). Surface proteins regulate the synthesis, deposition, and degradation of triglycerides in LDs. The synthesis, deposition, and degradation of triglycerides in LDs are regulated by lipid droplet surface proteins, and the dynamic changes of surface proteins are regulated by multiple transcription factors (Olzmann & Carvalho, 2019).

There is a strong association between low vitamin D level and obesity. Obese individuals generally have low vitamin D levels (Vimaleswaran et al., 2013; Wakayo et al., 2016). In addition, vitamin D and its metabolites can regulate adipocyte physiology and pathophysiology. For example, 1,25(OH)_2_D_3_ can inhibit adipocyte fat storage (Chang & Kim, 2016; Larrick et al., 2018), increase the expression and activity of lipolysis (Vu et al., 1996), induce adipocytes apoptosis (Sergeev, 2009), and block adipocyte differentiation by inhibiting the action of PPARγ in 3 T3‐L1 cells (Kong & Li, 2006). PPARγ, a group of transcription factors, is involved in the surface protein regulation of LDs (Rodriguez & Kersten, 2017), and is an essential factor in adipocyte differentiation expressed in adipose progenitor cells around the blood vessels of white adipose tissue (Tang et al., 2008). An in vivo study showed that PPARγ‐knockout mice demonstrate systemic adipose tissue atrophy (Sardella et al., 2018), with mice surviving only for few days (Imai et al., 2004), thus indicating that PPARγ is necessary for the survival of mature adipocytes.

However, most studies on vitamin D‐regulating action of PPARγ on obesity have focused on adipocyte differentiation. Our hypothesis was that VitD3 inhibits adipocyte hypertrophy by suppressing the expression of PPARγ in white adipocytes. To investigate this hypothesis, one separate interventional study with VitD3 diet was performed to examine the effect of VitD3 in white adipocytes in C57BL/6J mice with obesity. This study provides a basis for elucidating the effect of VitD3 on adipocyte hypertrophy in DIO mice and provides a new idea for its effective dosage treatment of the obese.

## 2 METHODS AND MATERIALS

### 2.1 Animals and diets

Male 5‐week‐old C57BL/6J mice were purchased from Beijing Huafukang Biotechnology Co., Ltd, Beijing, China. Mice were raised in controlled conditions with a temperature range of 19–26°C, relative humidity ranging between 40 and 70%, 12:12 h of light/dark cycle, and ad libitum access to standard laboratory chow diet and water. After 1 week, mice were randomly assigned into two groups: normal diet group (CON, *n* = 18) and high‐fat diet group (HFD, *n* = 108), providing either 10% (3.6 kcal/g) or 60% (5.1 kcal/g) of total energy as fat, respectively (TP23302 and TP23300, respectively, TROPHIC Animal Feed High‐tech Co. Ltd. Nantong, Jiangsu, China). After 10 weeks, normal diet mice were continued to be fed after excluding the weight extremes (*n* = 14), and high‐fat diet mice with a body weight (single mouse) of 20% (Buettner et al., 2007; Guo et al., 2020; Pang et al., 2022) or more of the mean body weight of CON group were selected for further studies. Compositions of the normal diet and high‐fat diet are listed in Table 1.

**TABLE 1:** Compositions of the normal diet and high‐fat diet.

	High‐fat diet (TP23300)	Normal diet (TP23302)
Ingredients (g/kg)		
Protein (casein, l‐cystine)	276	194
Carbohydrate (dextrin, sucrose)	250	—
Carbohydrate (dextrin, sucrose, corn) starch)	—	673
Fat (soybean oil, lard)	341	40
Fiber (Cellulose)	68	48
Mineral and vitamin mixture	65	45
Antioxidant (TBHQ)	0.07	0.01
Total	1000	1000
Macronutrient metabolizable energy (%)		
Protein	19	19
Carbohydrate	21	71
Fat	60	10

### 2.2 Dosage information

The obese mice treatment groups included the low‐dose VitD3 group (HFD + VDL + DMSO, 5650 IU/kg·food), medium‐dose VitD3 group (HFD + VDM + DMSO, 8475 IU/kg·food), high‐dose VitD3 group (HFD + VDH + DMSO, 11,300 IU/kg·food), PPAR γ antagonist group (HFD + GW9662, 2 mg/kg BW) (Bahi et al., 2014; Guo et al., 2017), and solvent group (HFD + DMSO).

The VitD3 dosage was based on previous studies; the VDH was 100,00 IU/kg (Lee et al., 2018; Song & Sergeev, 2015) (ten times higher than the recommended level of 1000 IU/kg food; Nuclear Regulatory Commission (NRC), 1995), the VDL was 5000 IU/kg (Seldeen et al., 2017), and the VDM was 7500 IU/kg according to the principle of isometric difference. The feed will be sterilized by irradiation in barrier system before leaving the factory, previous studies have found that irradiation sterilization results in approximately 13% loss of VitD3 (Li et al., 2019). Therefore, the final dose was as mentioned above. GW9662 (Cayman, Item No. 70785, CAS No. 22978‐25‐2) dissolved in 1% DMSO was intraperitoneally administered to high‐fat diet mice every other day (10 mL/kg·BW).

**FIGURE 1:**
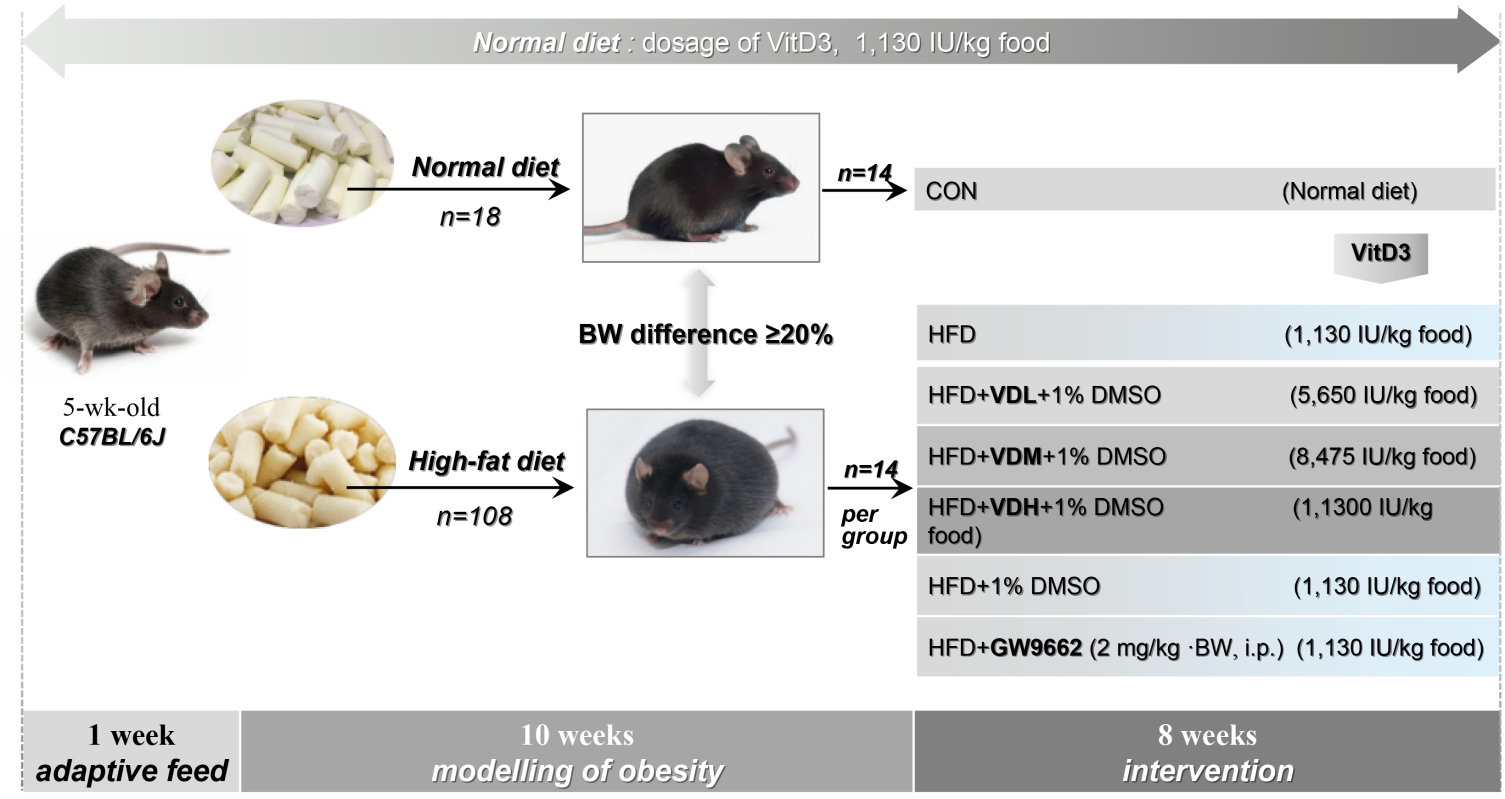
Grouping and experimental procedure. The raw dosage of VitD3, VDL was 5650 IU/kg, VDM was 8475 IU/kg, and VDH was 11,300 IU/kg. Based on that barrier system irradiation sterilization results in approximately 13% loss of VitD3, the final dosage is as given here. GW9662 and 1% DMSO were intraperitoneally (i.p.) administered to high‐fat diet mice.

The weight was measured weekly, and energy intake was calculated per group based on the amount of food intake by the animals and their caloric equivalence. After 8 weeks of intervention, mice were fasted the night before blood and tissue collection, then anesthetized with phenobarbital sodium (50 mg/kg·BW, Animal Veterinary Medical Association Guidelines for the Euthanasia of Animals: 2020 Edition), and executed. Body length (cm) was measured to calculate the Lees index [(weight)^⅓^/body length × 1000]. The epididymal (epiWAT, visceral), inguinal (ingWAT, subcutaneous), perirenal, and brown fat were weighed. After blood collection by cardiac puncture, serum was collected for 25(OH)D levels and lipid profile assays. EpiWAT was used for real‐time quantitative polymerase chain reaction (RT‐qPCR) and western blot, or fixed in 4% paraformaldehyde for histological analysis. All animal studies were approved by the Southwest Medical University Ethics Committee (approval number: 2020716) (Figure 1).

### 2.3 Biochemical parameters

Serum TG, total cholesterol (TC), low‐density lipoprotein cholesterol (LDL‐C), and high‐density lipoprotein cholesterol (HDL‐C) levels were determined by routine laboratory methods using an automatic biochemical analyzer (Mindray, Shenzhen, China).

### 2.4 White adipose tissue histology analysis

EpiWAT was fixed in 4% paraformaldehyde overnight, embedded in paraffin, sectioned (5 μm), and stained with hematoxylin and eosin (H&E). Digital images were acquired using a digital tricamera microscope BA210DigitaL (Motic, Hong Kong, China). Four mice were selected for each group, and three images of each mouse were captured for the following analysis. The general lesion was observed at 40× magnification for each slice, and photos were taken at 200× magnification to observe the specific lesion. The size of the adipocyte area was determined using the Image‐Pro Plus 6.0 (Media Cybernetics, Rockville, MD, USA).

### 2.5 Serum 25(OH)D

Body VitD3 level was evaluated using a mouse 25(OH)D ELISA kit (E‐EL‐0015c, Elabscience, Beijing, China) according to the manufacturer's instructions. The detection limit of this kit was 3.13–200 ng/mL.

### 2.6 Real‐time quantitative polymerase chain reaction (RT‐qPCR)

RNA was isolated from epiWAT using an Animal Total RNA Isolation Kit (RE‐03011, FOREGENE, Chengdu, Sichuan, China) according to the manufacturer's protocol. cDNA was synthesized from 500 ng of total RNA using the PrimeScript™ RT Reagent Kit (RR047A, TAKARA, Maebashi, Japan). The reaction was performed at 37°C for 15 min, followed by inactivation of reverse transcriptase at 85°C for 5 s. The PCR parameters were predenatured at 95°C for 30 s, followed by 40 cycles of denaturation at 95°C for 10 s, and annealing at 60°C for 30 s. The comparative CT method was used to determine the target amount, normalized to an endogenous reference Glyceraldehyde 3‐phosphate dehydrogenase (GAPDH), and relative to a calibrator (2^−△△ct^). All RT‐qPCR experiments were performed in triplicate. The primers used are listed in Table 2.

**TABLE 2:** Primers used for Real‐time Quantitative Polymerase Chain Reaction (RT‐qPCR).

ID	Gene	Forward sequence (5′→3′)	Reverse sequence (3′→5′)
14,433	*Gapdh*	5′‐ACCTGCCAAGTATGATGA‐3′	3′‐GGAGTTGCTGTTGAAGTC‐5′
66,853	*Atgl*	5′‐GACAGCTCCACCAACATCCA‐3′	3′‐GAGGCGGTAGAGATTGCGAA‐5′
14,311	*Fsp27*	5′‐CAGAAGCCAACTAAGAAGA‐3′	3′‐ATAGGAAAGCGAGTATGTG‐5′
19,016	*Ppar γ*	5′‐ATCTCCACCTTATTATTCTGAA‐3′	3′‐TCAATCGGATGGTTCTTC‐5′
11,770	*Fabp4*	5′‐AGGTGAAGAGCATCATAAC‐3′	3′‐CGCCTTTCATAACACATTC‐5′

Abbreviations: ATGL, adipose triglyceride lipase; FABP4, fatty acid‐binding protein 4; Fsp27, fat‐specific protein 27; PPAR γ, peroxisome proliferator‐activated receptor γ.

### 2.7 Western blot analysis

First, approximately 100 mg of epiWAT was weighed, mixed with RIPA lysis buffer (BioTeke, PP1902), and homogenized with low temperature, supernatant was centrifuged and extracted, and protein concentration was determined with bicinchoninic acid method (BioTeke, PP1002). Proteins were separated by 10% sodium dodecyl sulfate–polyacrylamide gel electrophoresis (SDS‐PAGE) (P1200, Solarbio, China) and transferred to polyvinylidene fluoride (PVDF) membrane (ISEQ00010, Millipore, USA). The membrane was blocked with 5% nonfat milk (P0216‐300 g, Solarbio, China) containing 0. 1% Tween® 20 (T8220, Solarbio, China). After washing with 1% Tris‐buffered saline with Tween® 20 (TBST), the membranes were incubated with specific primary antibodies overnight. After being rinsed in TBST, the membranes were incubated with anti‐rabbit immunoglobulin G (IgG) and horseradish peroxidase (HRP)‐linked antibody diluted at 1:2000 (7074S, CST, USA) for 1 h. The protein expression was detected by GelView system (6000 plus, BLT, China), and immunoreactive bands were detected by ImageJ (National Institutes of Health and the Laboratory for Optical and Computational Instrumentation, CA, USA). The specific primary antibodies were against ATGL (CST, 2439S), Fsp27 (ab213693), PPARγ (Abcam, ab209350), and FABP4 (CST, 2120S). β‐actin (4970 T) and GAPDH (Bioss, bs‐10900R) were used as an internal control. All experiments were repeated three times, and mean values were derived.

### 2.8 Statistical analysis

Data are presented as the mean ± SD. Statistical differences between two and five groups were determined by Student's *t*‐test or one‐way analysis of variance (ANOVA) following the Dunnett's multiple comparison method. The assumption of normality was tested using the Shapiro–Wilk test. All statistical tests were two‐tailed, and the level of significance was established at *p <* .05 using SPSS software (version 25; IBM Corporation, NY, USA).

## 3 RESULTS

### 3.1 Obese mice model

#### 3.1.1 Body weight and food intake energy

**FIGURE 2:**
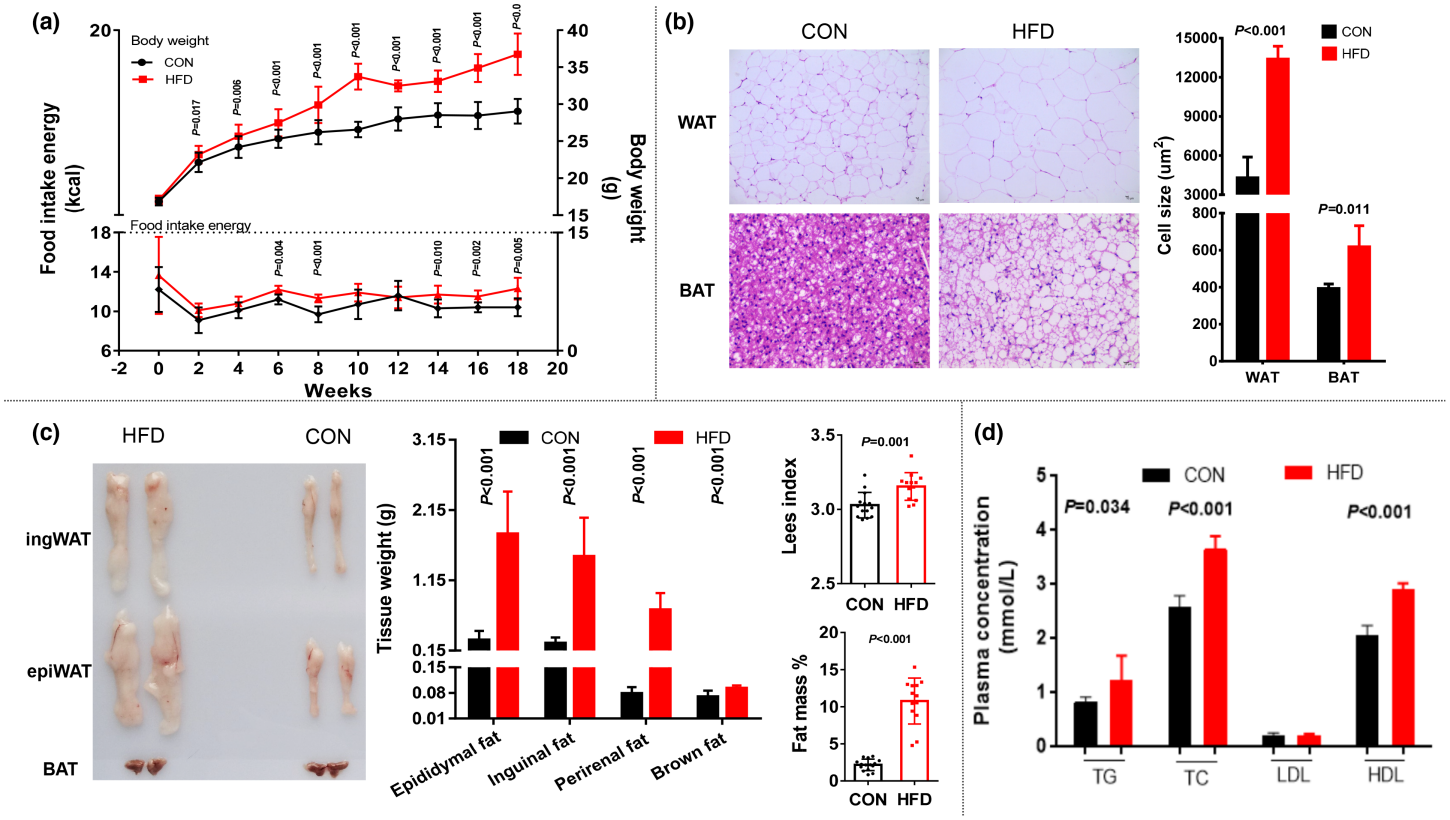
Obese mice model. (a) Body weight (upper panel) and food intake energy (below panel) of CON and HFD group from week 1 to week 18. (b) Left: Representative H&E images of epiWAT, and BAT in CON and HFD group (scale bar, 10 μm; magnification, 200×). Right: Cell size of epiWAT and BAT adipocytes in CON and HFD group. (c) Representative photographs of fat pads (epiWAT, ingWAT, and BAT) (left) and quantification (middle), Lees index, and fat mass rate (total fat/body weight × 100%) analysis of CON and HFD group. (d) Serum lipid profiles (TG, TC, LDL, and HDL) in CON and HFD groups. Data are represented as mean ± SD. Comparison of CON vs. HFD. (*n* = 14 per group), significance was determined by Student's *t*‐test analysis, *p < .05.

The body weight of the HFD group was higher than that of the CON group after the second week. Compared with the average body weight of the CON group, body weight in 77.57% (83/107) of mice in the HFD group increased by more than 20% by the 10th week. Body weight difference between the two groups was 26.50% in the 10th week and 26.91% in the 18th week, implying that the obesity model was stable during the intervention period (11–18th week) (Figure 2a). The food intake of mice was recorded during the entire experimental period, and the HFD group consumed more than the CON group (Figure 2a).

#### 3.1.2 Indexes of obesity

The fat mass rate, Lees index, and the weight of inguinal, epididymal, perirenal, and brown fat in the HFD group were higher than those in the CON group. The fat mass rate in the HFD group was approximately five times higher than that in the CON group, indicating that the increase in adipose tissue mainly caused weight gain in obese mice. The Lees index of mice in the HFD group reached 313, which can be regarded as an obese animal model index (Hariri & Thibault, 2010) (Figure 2c). The TG, TC, and HDL concentrations were increased in the plasma of the HFD group, while the low‐density lipoprotein (LDL) level did not change (Figure 2d). Compared with the CON group, the cell size of white and brown adipocytes in the HFD group was markedly increased by approximately three times. Consequently, the increase in fat mass in adult mice was mainly caused by an increase in white adipocyte volume (Figure 2b).

### 3.2 The effect of VitD3 on obese mice

#### 3.2.1 The change of physical index and plasma lipid assays on obese mice with VitD3 intervention

**FIGURE 3:**
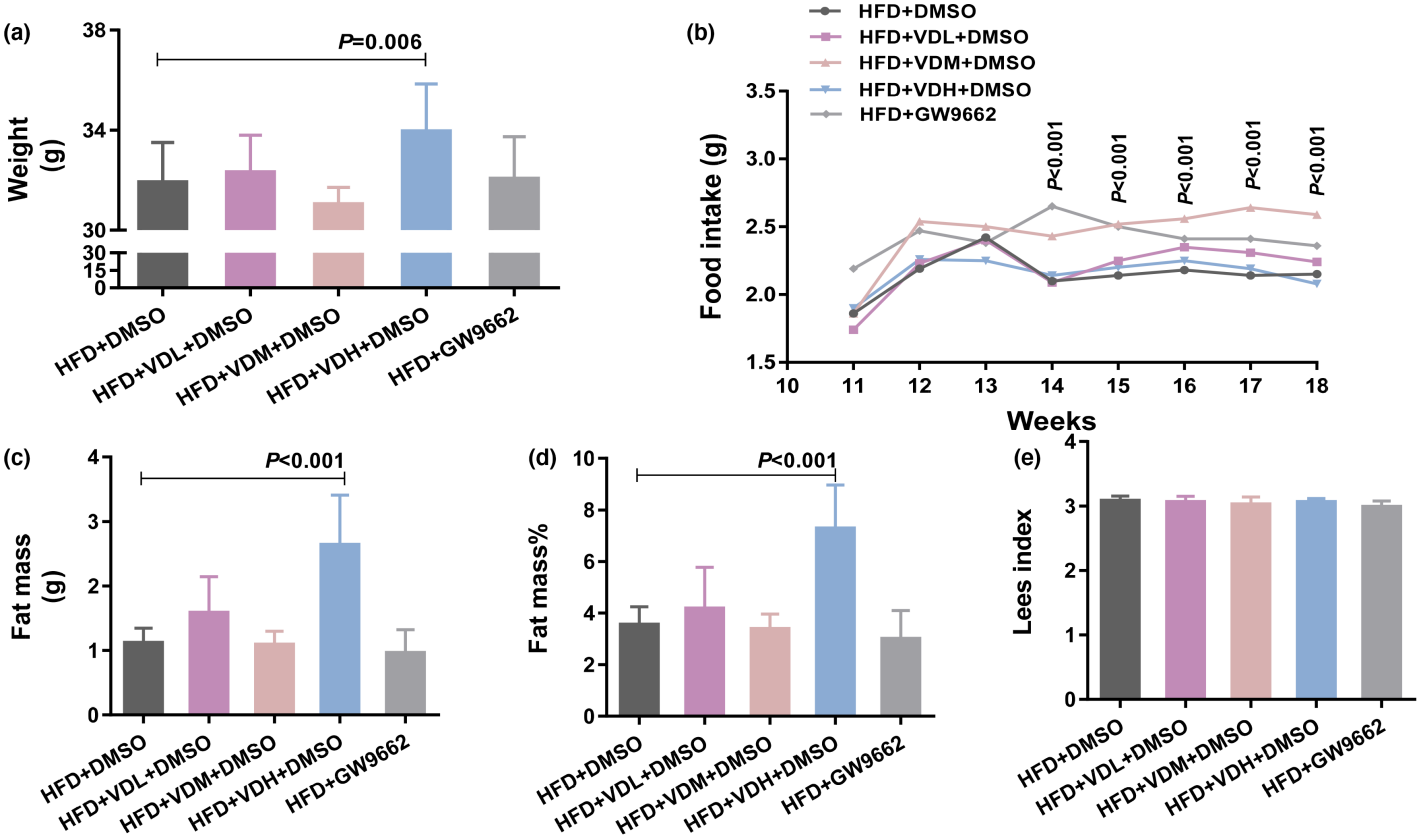
The change of physical index on obese mice with VitD3 intervention. (a) Body weight of five intervention groups at 18 weeks. (b) Food intake of five intervention groups from week 11 to week 18. (c) Fat mass of five intervention groups. (d) Fat mass rate of five intervention groups. (e) Lees index of five intervention groups. Data are expressed as the mean ± SD (*n* = 14 per group), analyzed by one‐way ANOVA following Dunnett's multiple comparisons. *p < .05, as compared with the HFD + DMSO group.

**FIGURE 4:**
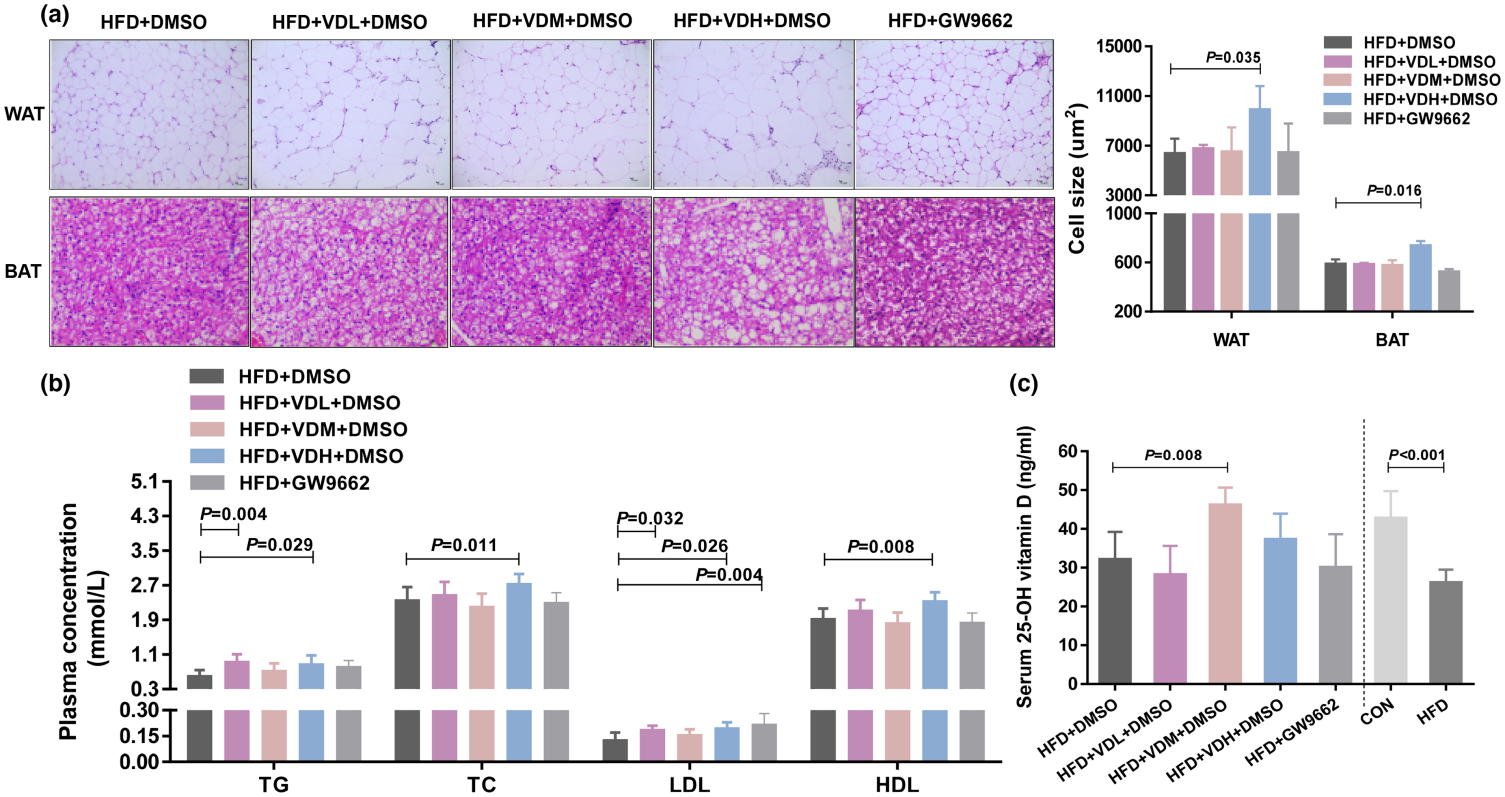
The change of adipocyte size and plasma lipid assays on obese mice with VitD3 intervention. (a) Left: Representative H&E images of epiWAT, and BAT in five intervention groups (scale bar, 10 μm; magnification, 200×). Right: Cell size of epiWAT and BAT adipocytes (*n* = 4–6 per group). (b) Serum lipid profiles in five intervention groups (*n* = 14 per group). (c) Serum concentrations of 25(OH)D in five intervention groups (*n* = 14 per group). Data are expressed as the mean ± SD, analyzed by one‐way ANOVA following Dunnett's multiple comparisons. *p < .05, as compared with the HFD + DMSO group. Comparison of CON vs. HFD, significance was determined by Student's *t*‐test analysis, ^#^ *p* < .05.

The body weight of the VDH group increased (Figure 3a), which was not related to food intake (Figure 3b, Table 3) and was presumed to be caused by high‐dose VitD3 intake (Figure 3c, Table 4). Although the food intake (Figure 3b, Table 3) and VitD3 intake (Figure 3c, Table 4) of the VDM group increased, body weight did not change (Figure 3a). The adipose tissue mass and fat mass rate of the VDH group increased (Figure 3d,e). No modification of the Lees index was observed after intervention. Therefore, weight gain in the VDH group was contributed by an increase in adipose tissue mass, that is, the intake of high‐dose VitD3 led to an increase in fat mass. Furthermore, the VDH group showed higher TC, TG, LDL, and HDL levels than solvent control. The VDL group had higher TG and LDL levels (Figure 4b). Overall, these data suggest that vitamin D3 supplementation aggravates obesity and promotes lipid metabolism disorders.

**TABLE 3:** Daily food intake in obese mice (g/day, $\bar{x}$± s).

Week	HFD + DMSO	HFD + DMSO + VDL	HFD + DMSO + VDM	HFD + DMSO + VDH	HFD + GW9662	*F*	*p*
11	1.86 ± 0.22	1.74 ± 0.33	1.86 ± 0.32	1.90 ± 0.26	2.19 ± 0.30	2.356	.076
12	2.19 ± 0.55	2.23 ± 0.25	2.54 ± 0.20	2.26 ± 0.19	2.47 ± 0.47	1.303	.291
13	2.42 ± 0.38	2.40 ± 0.30	2.50 ± 0.37	2.25 ± 0.34	2.38 ± 0.42	0.407	.802
14	2.10 ± 0.16	2.09 ± 0.14	2.43 ± 0.18*	2.14 ± 0.10	2.65 ± 0.37*	8.141	<.001
15	2.14 ± 0.07	2.25 ± 0.11	2.52 ± 0.11*	2.20 ± 0.13	2.50 ± 0.09*	18.288	<.001
16	2.18 ± 0.06	2.35 ± 0.10*	2.56 ± 0.09*	2.25 ± 0.04	2.41 ± 0.09*	21.149	<.001
17	2.14 ± 0.13	2.31 ± 0.10*	2.64 ± 0.16*	2.19 ± 0.13	2.41 ± 0.15*	14.125	<.001
18	2.15 ± 0.13	2.24 ± 0.10	2.59 ± 0.15*	2.08 ± 0.10	2.36 ± 0.15*	14.714	<.001

*Note*: Data are expressed as the mean ± SD (*n* = 14 per group) and analyzed by one‐way ANOVA following Dunnett's multiple comparisons.

* *p* < .05, compared with the HFD + DMSO group.

**TABLE 4:** Daily VitD3 intake in obese mice(IU, $\bar{x}$ ± s).

Week	HFD + DMSO	HFD + DMSO + VDL	HFD + DMSO + VDM	HFD + DMSO + VDH	HFD + GW9662	F	*p*
11	2.10 ± 0.25	9.82 ± 1.87	15.77 ± 2.68	21.45 ± 2.93*	2.47 ± 0.34	127.43	<.001
12	2.47 ± 0.62	12.59 ± 1.41	21.50 ± 1.66	25.54 ± 2.11*	2.79 ± 0.53	394.78	<.001
13	2.52 ± 0.28	13.54 ± 1.72	21.15 ± 3.10	25.46 ± 3.84*	2.69 ± 0.48	119.82	<.001
14	2.37 ± 0.18	11.83 ± 0.80	20.61 ± 1.51	24.14 ± 1.15*	3.00 ± 0.42	674.97	<.001
15	2.42 ± 0.08	12.72 ± 0.63	21.34 ± 0.93	24.84 ± 1.44*	2.82 ± 0.10	965.82	<.001
16	2.47 ± 0.07	13.25 ± 0.58	21.68 ± 0.72	25.37 ± 0.49*	2.73 ± 0.10	3403	<.001
17	2.42 ± 0.15	13.02 ± 0.60	22.34 ± 1.33	24.70 ± 1.52*	2.73 ± 1.64	881.33	<.001
18	2.43 ± 0.15	12.67 ± 0.52	21.98 ± 1.29	23.46 ± 1.12*	2.66 ± 0.17	791.35	<.001

*Note*: Data are expressed as the mean ± SD (*n* = 14 per group) and analyzed by one‐way ANOVA following Dunnett's multiple comparisons.

* *p* < .05, compared with the HFD + DMSO group.

#### 3.2.2 The change of adipocyte size on obese mice with VitD3 intervention

An increase in adipose tissue mass can be attributed to an increase in adipocyte size or number due to abnormal differentiation, or both. Therefore, adipocyte size in the adipose tissue was measured to reveal the mechanism of increased adiposity in VitD3 intervention mice. H&E staining indicated that white and brown adipocytes were larger in the VDH group than in the solvent control (Figure 4a).

#### 3.2.3 The change of serum 25(OH)D on obese mice with VitD3 intervention

The HFD mice displayed a reduction in the 25(OH)D serum level compared to CON mice. Moreover, in mice fed with a VitD3 diet, serum 25(OH)D levels were different. The VDM group had an elevated 25(OH)D concentration than the solvent control, but there were no significant differences compared with the CON group. Low and high doses of VitD3 did not affect the serum 25(OH)D levels in obese mice, thus demonstrating that dietary VitD3 might be affecting the systemic vitamin D status in obese mice (Figure 4c, Table 5).

**TABLE 5:** The effect of vitamin D3 on serum 25(OH)D_3_ in obese mice (ng/mL, $\bar{x}$ ± s).

Group	25(OH)D_3_
CON	42.71 ± 7.01
HFD	26.07 ± 3.42*
HFD + DMSO	32.08 ± 7.14
HFD + VDL + DMSO	28.12 ± 7.50
HFD + VDM + DMSO	46.12 ± 4.51**
HFD + VDH + DMSO	37.21 ± 6.72
HFD + GW9662	30.03 ± 8.62
*F*	6.223
*p*	.002

*Note*: Data are expressed as the mean ± SD (*n* = 14 per group). Intervention groups were analyzed by one‐way ANOVA followed by Dunnett's multiple comparisons.

* *p* < .05.

** *p* < .05, as compared with the HFD + DMSO group. Comparison of CON vs. HFD, significance was determined by Student's *t*‐test analysis.

#### 3.2.4 The change of adipocyte‐related gene on obese mice with VitD3 intervention

**FIGURE 5:**
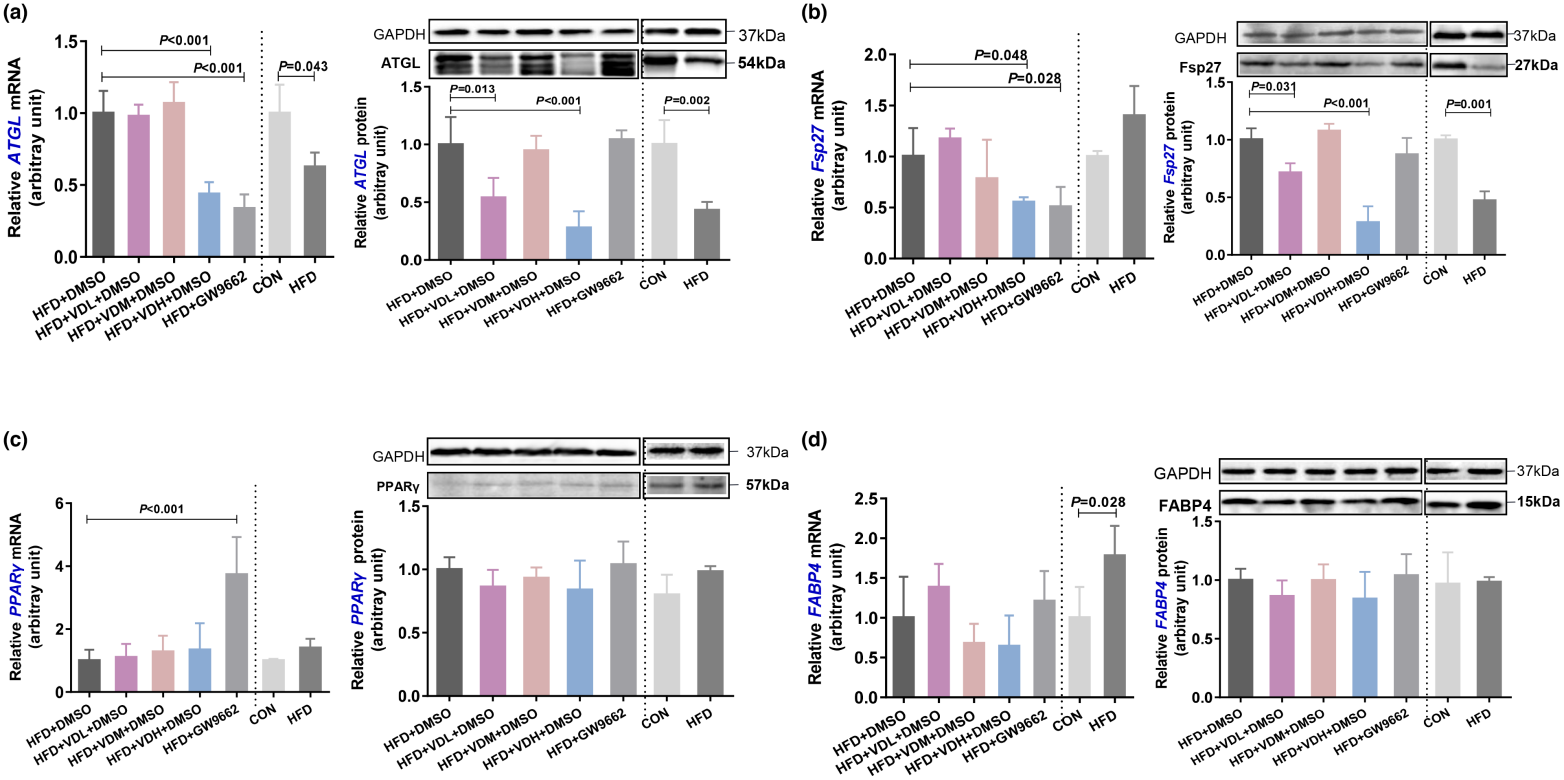
The change of adipocyte‐related gene on obese mice with VitD3 intervention. Protein (right: Western blot) and mRNA (left: RT‐qPCR) expression level of ATGL (a), Fsp27 (b), PPAR γ (c), FABP4 (d) of epiWAT in all groups (*n* = 4–6 per group). All experiments were repeated at least three times. Data are expressed as the mean ± SD. Intervention groups are analyzed by one‐way ANOVA following Dunnett's multiple comparisons, *p < .05, as compared with HFD + DMSO group. Comparison of CON vs. HFD, significance was determined by Student's *t*‐test analysis, ^#^ *p* < .05.

The mRNA and protein levels of lipid metabolism‐associated markers in adipose tissue were measured by RT‐qPCR and western blotting to explore the specific mechanism of VitD3 intervention causing adipocyte hypertrophy. Adipose triglyceride lipase (ATGL), a lipolysis‐associated gene, was overexpressed in CON group than in HFD group, and was significantly suppressed by low‐ and high‐dose VitD3 interference compared with solvent control (Figure 5a). The fat‐specific protein 27 expressions (Fsp27), undertaking the large unilocular lipid droplet formation in white adipocytes, was lower in the HFD group than in the CON group. However, low and high doses of VitD3 decreased the Fsp27 expression (Figure 5b). To determine whether the high‐fat mass was caused by adipocyte hyperplasia, the peroxisome proliferator‐activated receptor γ (PPARγ), an essential adipocyte differentiation transcription factor was measured. The PPARγ expression in the HFD and CON groups was not different in adult mice, and its level did not change in the VitD3 intervention group (Figure 5c). Adipocyte‐specific fatty acid‐binding protein (FABP4), which acts as a fatty acid chaperone that couples intracellular lipids to biological targets and signaling pathways, was measured. There was no significant difference in FABP4 gene expression profiles between the groups (Figure 5d).

## 4 DISCUSSION

Based on the present study results, we rejected the hypotheses that VitD3 inhibits the growth of adipocyte size by suppressing PPARγ expression in white adipocytes of obese mice. Conversely, VitD3 intervention increased body weight, fat mass, and blood lipid levels in obese mice, possibly via inhibiting the ATGL expression.

The prevalence of excess body weight has been increasing worldwide since the 1970s. In 2016, approximately 40% of adults were obese (Sung et al., 2019). Therefore, safer and potent therapeutics are required to mitigate the problem, necessitating the continued use of animal models to discover, validate, and optimize novel therapeutics for use in humans. The use of a model of diet‐induced obesity in animals is effective for studying the physiopathology of complications associated with obesity, as it is the closest template to the genesis of obesity in humans (Hariri & Thibault, 2010; Kleinert et al., 2018). The inbred C57BL/6J mouse strain is widely used as a model for (DIO‐induced obesity) since it is prone to developing severe obesity, elevated adiposity, glucose intolerance, and moderate insulin resistance. Male mice are more susceptible to DIO than females. Male mice develop obesity earlier and in severe forms than female mice. According to previous studies, mice take 8–12 weeks to develop obesity when their diet contains 40–60% energy from fat (Kleinert et al., 2018).

Furthermore, If mice are fed on an HFD diet at an early stage (<8 weeks old), the development of obesity and adiposity is markedly pronounced (Fernandes et al., 2016). In this study, we selected 5‐week‐old C57BL/6J male mice fed on a 60% fat diet for 18 weeks, and the body weight differences (26.5–26.91%) were stable within 10–18 weeks. Moreover, blood lipids and body mass were significantly higher in than in normal diet mice, indicating that a high‐fat diet contributes to severe lipid metabolic disturbances.

Low circulating level of total and free 25(OH)D, indicating low vitamin D concentration, is associated with obesity in humans (Earthman et al., 2012; Feghaly et al., 2020). For vitamin D to become biologically active, it has to be converted into 25‐hydroxyvitamin D (25(OH)D) in the first hydroxylation step catalyzed by four enzymes (CYP2R1, CYP27A1, CYP2J6, and CYP3A11). A second hydroxylation step catalyzed by CYP2B1 in the kidneys produces 1,25‐dihydroxy vitamin D (1,25(OH)_2_D_3_), the active form of cholecalciferol, a potent activator of the vitamin D receptor (VDR). Furthermore, 25(OH)D and 1,25(OH)_2_D_3_ can be catabolized by 24‐hydroxylase CYP24A1 to generate inactive metabolites (Saponaro et al., 2020). In addition, several studies have shown that the free forms of 25(OH)D and 1,25(OH)_2_D_3_ decreased during obesity (Liu et al., 2016; Walsh et al., 2017; Xiao et al., 2020). Many researchers found that 1,25(OH)_2_D_3_ inhibits 3 T3‐L1 preadipocyte differentiation (Kong & Li, 2006), decreases TG accumulation (Chang & Kim, 2016), increases lipid oxidation (Larrick et al., 2018), and promotes lipolysis (Chang & Kim, 2016) in mature 3 T3‐L1 adipocytes.

The results showed that mice fed a high‐fat diet with a high level of VitD3 (5000 IU and 10,000 IU/kg) had increased body weight and fat mass and increased adiposity markers (plasma concentrations of TG, TC, HDL, and LDL). Furthermore, the VitD3 intervention group mice were more susceptible to the development of HFD‐induced obesity through adipocyte hypertrophy, attributed to impaired lipolysis. In this study, the regulation of lipolysis by VitD3 was mediated by the inhibition of ATGL, responsible for the initial step of TG degradation (Cerk et al., 2018). Our previous research indicated that the mRNA expression of ATGL increased after 1,25(OH)_2_D_3_ intervention in 3 T3‐L1 adipocytes in a hypertrophy model (Xiang et al., 2020). In the present study, serum 25(OH)D levels did not increase in obese mice after 8 weeks of VitD3 intervention, so it is assumed that the 1,25(OH)_2_D_3_ levels in obese mice did not increase either. Labban et al. reported that VitD3 and its metabolites could accumulate in adipose tissue, and VitD3 had the highest ability of deposition in WAT, hindering the bioavailability of vitamin D and indirectly decreasing the serum level of 1,25(OH)_2_D_3_ (Labban et al., 2020). Nandan et al., treated obese rats through intragastric administration of 1,25(OH)_2_D_3_, their results showed that 1,25(OH)_2_D_3_ inhibited an increase in body weight and decreased fat mass in obese rats (Nandan et al., 2018). Collectively, our results demonstrated that obese mice intervened by VitD3 did not generate 1,25(OH)_2_D_3_ adequately, and VitD3 intervention inhibited the expression of ATGL in obese mice WAT. However, the detailed mechanism could not be elucidated.

Meanwhile, as the adipocytes of obese mice treated with VitD3 tend to be hypertrophic, the expression of Fsp27 (Chen et al., 2020), an integral part of LD proteins required to form a large unilocular LD in adipocytes, relatively decreased. Moreno‐Navarrete et al. reported that Fsp27 expression was negatively associated with BMI and fat mass rate in visceral adipose tissues (Moreno‐Navarrete et al., 2014). Fsp27 stores fat within adipose tissue, preventing spillover or ectopic accumulation in nonadipose tissue. Thus, lack of Fsp27 in adipose tissue causes the breakdown of fat, causing health issues typically associated with obesity (Karki, 2019).

In addition, the PPARγ antagonist GW9662 did not inhibit the PPARγ protein level in WAT of obese mice, which might be attributed to the inadequate duration of intervention. Furthermore, VitD3 did not affect the PPARγ expression in the WAT of obese mice. Few researchers have focused on changes in the PPARγ gene expression in animal and human trials in obese conditions, probably because PPARγ is a transcription factor that participates in preadipocyte differentiation. Several studies have demonstrated that 1,25(OH)_2_D_3_ can inhibit PPARγ protein levels at an early time point in 3 T3‐L1 mature adipocytes (Ji et al., 2015; Nobre et al., 2018). In conclusion, PPARγ is required for mature adipocytes or obese mice (Imai et al., 2004), but at the same time, our results show that PPARγ expression does not differ by degree of obesity in adulthood and that obesity due to VitD3 is not achieved by affecting PPARγ expression. FABP4 acts as a fatty acid chaperone (Furuhashi & Hotamisligil, 2008), which couples intracellular lipids to biological targets and signaling pathways and contains peroxisome proliferator response elements (PPREs) regulated by PPARγ (Tontonoz & Spiegelman, 2008). Our results demonstrated that VitD3 did not influence fatty acid transportation, parallel to the change in PPARγ. Chang et al. found that 1,25(OH)_2_D_3_ (24 h, 100 nmol/L) significantly decreased FABP4 and PPARγ mRNA expression by 43.9% and 49.2%, respectively, in differentiated 3 T3‐L1 adipocytes (Chang & Kim, 2016). Further studies demonstrated that 1,25(OH)_2_D_3_ did not influence fatty acid transportation in differentiated 3 T3‐L1 adipocytes. Therefore, we believe that 1,25(OH)_2_D_3_ can inhibit the expression of FABP4 only at the transcriptional level in 3 T3‐L1 mature adipocytes.

For the dose of VitD3, as far as humans are concerned, according to the body size coefficient method (Wei et al., 2010), the dose of VitD3 in obese mice in this experiment was extrapolated to the corresponding dose range of 2112–5355 IU (see Tables S1 and S2) in people with mild to severe obesity, and the Endocrine Society Clinical Practice Guideline suggest a higher dose (two to three times higher; at least 6000–10,000 IU/d) of vitamin D to treat vitamin D deficiency to maintain a 25(OH)D level above 30 ng/mL, followed by maintenance therapy of 3000–6000 IU/d (Holick et al., 2011). In this experiment, the medium dose of VitD3 restored the serum 25(OH)D level to normal level in obese mice, corresponding to the dose of 3632–4416 IU in people with obesity, while the high‐dose group eventually led to the increase of obesity although the serum 25(OH)D level was not different from that of normal mice. In conclusion, for the treatment of vitamin D deficiency in people with obesity, we believe that it is more reasonable to intervene with maintenance doses (3000–6000 IU/d), regular observation of serum 25(OH)D levels, and individualized long‐term intervention programs than high‐dose shock therapy (6000–10,000 IU). Another, vitamin D and its metabolites have a variety of biological effects on the body and each has its own role, for obese patients with vitamin D deficiency, improving the conversion rate and utilization of vitamin D after intake into the body is the most critical, and the role of 1,25(OH)_2_D_3_ is only one of the many functions of vitamin D embodiment.

In conclusion, our study provides evidence that long‐term high‐dose VitD3 could increase the volume of adipocytes in obese mice by inhibiting the expression of ATGL. However, further studies are needed to elucidate how VitD3 inhibits the expression of ATGL, and we need more clinical trials to explore the intervention dose and duration of VitD3 in the treatment of VitD3 deficiency in obese patients.

## AUTHOR CONTRIBUTIONS

**Jingjing Zhang:** Conceptualization (equal); data curation (lead); formal analysis (lead); investigation (lead); methodology (lead); project administration (equal); writing – original draft (lead). **Yuanfan Zhang:** Methodology (equal); project administration (equal). **Yong Zhou:** Conceptualization (equal); methodology (equal); supervision (lead); validation (lead); writing – review and editing (lead). **Wenxin Zhao:** Methodology (equal); project administration (equal). **Jialu Li:** Methodology (equal); project administration (equal). **Dan Yang:** Methodology (equal). **Dian Xiang:** Methodology (equal). **Tingwan Du:** Methodology (equal). **Ling Ma:** Conceptualization (lead); funding acquisition (lead); methodology (equal); project administration (lead); resources (lead); supervision (lead); validation (lead); writing – review and editing (lead).

## FUNDING INFORMATION

This study was supported by the 2019 Science and Technology Project of Sichuan Province (2019YJ0483) and the 2019 National Innovation and Entrepreneurship Training Program for College Students (201910632042).

## CONFLICT OF INTEREST STATEMENT

The authors declare that they have no known competing financial interests or personal relationships that could have appeared to influence the work reported in this paper.

## Supporting information


Tables S1‐S2

## Data Availability

All data included in this study are available upon request by contact with the corresponding author.
