# Supplementary material for: Effect of vitamin D3 on lipid droplet growth in adipocytes of mice with HFD‐induced obesity
Source: Food Sci Nutr. 2023 Aug 24;11(10):6686–97. doi: 10.1002/fsn3.3618 (PMC10563741; doi:10.1002/fsn3.3618)
Supplement: Supplementary file 1 — Tables S1‐S2 [file FSN3-11-6686-s001.docx]

**Effect of vitamin D3 on lipid droplet growth in adipocytes of mice with HFD-induced obesity**

**Authors**: Jingjing Zhang^a^, Yuanfan Zhang^b^, Wenxin Zhao^b^, Jialu Li^b^, Dan Yang^b^, Lian Xiang^b^, Tingwan Du^b^, Yong Zhou^c*^ and Ling Ma^b*^

^a^ Department of Clinical Nutrition, Affiliated Hospital of Southwest Medical University, Luzhou, Sichuan, China.

^b^ Department of Nutrition and Food Hygiene, School of Public Health, Southwest Medical University, Luzhou, Sichuan, China.

^c^ Department of Medical Cell Biology and Genetics, School of Basic Medical Science, Southwest Medical University, Luzhou, Sichuan, China.

*Corresponding authors:

Ling Ma: xjml@swmu.edu.cn

**Supplementary materials**

| **Table 1.** Extrapolation to VitD3 dosage for obese people | | | | | | |
| --- | --- | --- | --- | --- | --- | --- |
| Group | VitD3 Dosage  (IU/kg·forage) | Food intake of obese mice in intervention period  (g/day) | VitD3 intake of obese mice  (IU/day) | Weight _a_  (g) | Weight _b_+20%  (IU) | Weight _b_+50%  (IU) |
| HFD+VD**L**+DMSO | 5000 | 2.25±0.20 | 11.25±1.0 | 32.36±1.32 | 2112~2390 | 2450~2774 |
| HFD+VD**M**+DMSO | 7500 | 2.52±0.22 | 18.9±1.65 | 33.23±3.19 | 3632~3806 | 4215~4416 |
| HFD+VD**H**+DMSO | 10000 | 2.22±0.24 | 22.2±2.4 | 33.51±2.22 | 4058~4614 | 4708~5355 |

Mice experimental data are expressed as the mean ± SD (n=14 per group). Dosage conversion according to the body size coefficient method, Dose (b, human)=Dose (a, mouse)×(k _b_ / k _a_)×(Weight _b_ / Weight _a_)^2/3^, k refers to the body size coefficient (Table 2.), k _a_=0.0899, k _b_=0.1057. Standard adult weight was set at 60 kg, Weight _b_+20%~30% is mild obesity, Weight _b_+30%~50% is moderate obesity, Weight _b_+50%~ is severe obesity.

| **Table 2.** Body size coefficients of different species of animals | | | | | | | | | |
| --- | --- | --- | --- | --- | --- | --- | --- | --- | --- |
| Animal | Mouse | Hamster | Rat | Guinea pig | Rabbit | Cat | Dog | Monkey | Human |
| Body size coefficient of mice | 0.0899 | 0.0862 | 0.086 | 0.092 | 0.1014 | 0.1086 | 0.1077 | 0.118 | 0.1057 |
